# Anti-Tuberculous Vaccines and Sera
1A paper read before the Bristol Medico-Chirurgical Society, Nov. 8th, 1905.


**Published:** 1906-03

**Authors:** J. M. Fortescue-Brickdale

**Affiliations:** Senior Physician to Out-Patients and Pathologist to the Royal Hospital for Sick Children, Bristol


"*Scire est nescire, nisi id me*
*Scire alius sciret.*"

MARCH, 1906.

INDEX THROUGH

### ANTI-TUBERCULOUS VACCINES AND SERA.[1]

BY

J. M. Fortescue-Brickdale, M.A., M.D. Oxon.,

*Senior Physician to Out-Patients and Pathologist to the Royal Hospital for Sick Children, Bristol.*

The general abandonment of Koch's first method of obtaining a specific immunising agent against tuberculosis has by no means acted as a check to further research along the same lines; on the contrary, if I may borrow a simile from the subject-matter of this paper, it seems to have acted like the injection of a toxin which produces a large number of bodies tending to neutralise its action. In fact, about twenty substances have been prepared since 1890, and more or less largely used. These fall into two great groups: the tuberculins which contain poisonous substances prepared from tubercle bacilli, and are intended to produce active immunity, and the anti-tuberculous sera, which, withdrawn from immunised animals, are intended to act as anti-toxins, and thus to secure a passive immunity.

[1] A paper read before the Bristol Medico-Chirurgical Society, Nov. 8th, 1905.

The tubercle bacillus when grown on artificial media produces very little in the way of soluble toxins or extra-cellular toxins; it can, however, by various methods be made to yield certain toxic substances, the chief of which are known as tuberculo-proteins and tuberculo-plasmins.

Tuberculo-proteins are fairly stable bodies, and can be extracted from media in which the bacilli are grown; the tuberculo-plasmins have to be obtained from actual bodies of the bacilli by some crushing process. Other substances are present in all tuberculins; and some of these undoubtedly contribute to the reaction produced, but it has not been shown that they are directly concerned in the immunising process.

*Tuberculins.*—Koch's old tuberculin (1890) is a glycerine extract of the bodies of the bacilli, together with some of the substances produced by them when grown on an artificial medium. The bacilli used are not virulent, but a long time is allowed for their growth and extraction, so that the preparation may be considered a powerful one. It is a sterilised and filtered preparation, and keeps well. It does not immunise animals against living cultures, but only against their metabolic products. Its action, as is well known, is local and general, and has been variously explained; it is obviously complicated, as it depends upon the presence of tuberculous foci in the body, and does not occur typically, at any rate in healthy subjects. Ehrlich[1] thought it due to some action on the intermediate zone of cells between the active foci and the healthy tissues. Marmorek[2] considered it a sensitiser of the bacilli, and not a true toxin. By its action he thought the tubercle bacilli were sensitised or stimulated to produce more toxin. Thus he explained the fact that it fails to produce symptoms in advanced cases, because then so much toxin is already being formed that no effect is produced by a comparatively small increase. Behring[3] has recently announced that its action is upon the product of the bacilli which he calls T.C., a remarkable substance capable of combining with and acting on the cells of the host. Wright[4]

[1] Ehrlich, quoted by Bosanquet, *Serums, Vaccines, and Toxines*, 1904.

[2] Marmorek, *Lancet*, 1903, ii. 1642. [3] Behring, *Ibid.*, 1905, ii. 1126.

[4] Wright, *Ibid.*, 1904, ii. 1138.

classes the active principle of tuberculin among the tuberculotropic bodies, that is bodies which produce within the tissues substances capable of combining with the tubercle bacilli.

Chemically the old tuberculin, which has been analysed by Hunter and others, contains proteins; it is now thought that the reactions are not specific for tuberculin, but may be produced by any of the bacterio-proteins, and even by allied bodies not derived from bacteria at all.[1]

The proteins are not the only substances contained in the old tuberculin. Hunter[2] found among other bodies alkaloids, and in 1891 he devised a method for separating the proteins or albumoses as he called them. These he injected instead of tuberculin, and claimed good results. A few cases were published in 1891 by himself and Watson Cheyne. Hahn's[3] method is similar to Hunter's; but if, as is now thought, the proteins are only carriers of the active principle, they would be useless in a perfectly pure form.

Klebs,[4] Ruck,[5] Denys,[6] and Spengler[7] have all devised methods for preparing tuberculins which may be considered as modifications of Koch's original tuberculin. Klebs' preparations (1894) are known as tuberculocidin and anti-phthisin (a concentrated form), and are mainly distinguished by being prepared by precipitation processes without the aid of heat. Ruck's (1896) is a watery filtered extract of bacilli, and Denys's (1902) is a similar preparation. Spengler's tuberculin (1904) is prepared from bovine bacilli, and is said to be less toxic than Koch's old tuberculin.

In 1897 Maksutow[8] prepared an extract from tuberculous tissues of guinea pigs, said to be capable of immunising experimental animals. It was thought to more nearly approach the real toxins of disease than preparations derived from artificial cultures.

[1] Krehl and Matthes, *Arch. f. exper. Path. u. Pharmakol.*, 1895, xxxvi. 437.
[2] Hunter, *Brit. M. J.*, 1891, ii. 169.
[3] Hahn, quoted by Batty Shaw, *Lancet*, 1905, i. 926.
[4] Klebs, quoted by Bosanquet, *op. cit.* [5] Ruck, *Therap. Gaz.*, 1896, xx. 308.
[6] Denys, quoted by Batty Shaw, *loc. cit.*
[7] Spengler, *Deutsche med. Wchnschr.*, 1904, xxx. 1129.
[8] Maksutow, quoted in *Brit. M. J.*, 1905, ii. 966.

The next group of tuberculins are not merely extracts, but are obtained by crushing the bacilli before extraction.

(1) In 1897 Koch[1] produced three new tuberculins, TO, TA and TR. Two are useless, namely TO, which is too weak and contains proteins, and TA, which excites suppuration at the site of injection. TR is a watery extract of dried and crushed bacilli, obtained by repeated centrifugalisations after TO has been separated off. It differs from tuberculins of the type of Koch's old tuberculin in containing tuberculo-plasmin, which produces only a general reaction and immunises against the bacilli themselves. It is prepared from virulent cultures, and may be heated to 60° C. for an hour with advantage.

(2) Koch's new tuberculin (1901) is an emulsion of crushed and dried bacilli. As it was occasionally found to contain living virulent organisms it is now heated to 60° C. for one hour. It acts similarly to TR, though thought to be more powerful. It does not keep well.

The two remaining tuberculins are:—

Béraneck's tuberculin[2] (1904), which is a mixture of two substances. The first, or basi-toxin, consists of the extra-cellular toxins of the bacilli cultivated on a special medium; the second, or acido-toxin, is a filtered extract prepared with orthophosphoric acid from the bodies of the bacilli after the basi-toxin has been removed. Its active principle is said to differ from the tuberculo-proteins of Koch's original preparation. It is said to immunise guinea pigs, and to be bactericidal *in vitro*.

Sciallero[3] (1904) states that he has obtained an extract from virulent cultures containing a fatty substance to which the peculiar staining properties of the bacilli are due. He claims to have immunised guinea pigs to virulent bacilli by this preparation.

I will now briefly consider—

I. The experimental evidence as to the tuberculins.

II. The clinical evidence as to their value.

III. The practical limitations to their usefulness.

[1] Koch, *Deutsche med. Wchnschr.*, 1897, xxiii. 209.

[2] Béraneck, *Brit. M. J.*, 1904, i. 735.

[3] Sciallero, *Il Policlin*, Nov. 1904; abstract in *Brit. M. J.*, 1905, i., Epitome, No. 48.

I. (1) In the first place all tuberculins have been shown to possess some immunising power on experimental animals. Some, as has been already noted, immunise animals only against themselves, or, in other words, induce toleration; but some have been shown to protect against virulent cultures of bacilli. It is not, of course, possible to say that this effect will also be produced in man, as no direct experiments have been made, but there is perhaps a presumption that it is so. Nor is it certain that tuberculin represents all the toxic products of the bacilli which cause the disease, and that immunisation against tuberculin is the same thing as immunisation against tuberculosis.

(2) Evidence of increased agglutinating power in the blood after injections of tuberculin (TR) has recently been brought forward by Wright[1] and others. This is taken as showing increased powers of resistance, but it is not always coincident with clinical improvement, which sometimes precedes any heightened agglutinative power in the blood serum.

(3) There is evidence that an increase of cells containing eosinophil granules in the blood is an indication of resistance to the invasion of the tubercle bacillus. Gianasso[2] found that among forty-eight children examined an increase in eosinophil cells was commoner in convalescent and improving cases; this is supposed to be due to increased activity in the bone marrow. Erben[3] has shown that in cases with marked anæmia great decrease in the activity of the bone marrow occurs. As far as I know this test has only been applied to Behring's new preparation, when it gave positive indications.

(4) Probably the most important indication of the immunising power of tuberculin is found in the increased opsonic index in the blood which Wright[4] and others have shown to occur. He and Urwick employed Koch's TR, and have shown that after injection the phagocytes are able to consume more tubercle bacilli than before, owing to the development of opsonins. This rise is, however, preceded by a fall in phagocytic power. Similar phenomena occur in auto-intoxication

[1] Wright, *loc. cit.* [2] Gianasso, *Brit. M. J.*, 1904, ii., Epitome, No. 217.
[3] Erben, *Ibid.*, 1905, ii., Epitome, No. 159. [4] Wright, *loc. cit.*

with bacillary products in the ordinary course of the disease.[1]

II. The clinical evidence of the value of tuberculin is not easy to express statistically; most of those who have used tuberculin think that it does good in selected cases, especially when combined with hygienic or "sanatorium" treatment. Pottenger constructed a table showing the results with and without tuberculin in patients at sanatoria; Batty Shaw, using Pottenger's table, added other cases, and I have been able to collect some further reports, as shown in the following table:—

SANATORIUM CASES TREATED WITH TUBERCULIN.

| AUTHOR. | REFERENCE. | CASES. | STAGE. | PERCENTAGE CURED. |
|---|---|---|---|---|
| Collected by—<br>Batty Shaw ... | *Lancet*, 1905, i. 922 ... | 656 | I. | 91 |
| Trudeau ... ... | q. Bosanquet, *Op. cit.* ... | 47 | I. | 41 |
| Turban ... ... | *Ibid.* ... ... ... ... ... | 86 | I. | 100 |
| Würtzen ... ... | *Tuberculosis*, (Leipzig), 1904, iii. 53. | 10 | I. | 100 |
| Sawyer ... ... | *Therap. Gaz.*, 1905, xxix. 364. | 14 | I. | 100 |
| Moeller ... ... | *B.M.J.*, Epit., 1904, ii. No. 249. | 1 | I. | 100 |
| Lœwenstein and Rappoport. | *Deutsche med. Woch.* vol. xxx. No. 23. | 48 | "Closed cases." | 17·3 |
| | | 862 | ... | 88 |

SANATORIUM CASES TREATED WITHOUT TUBERCULIN.

| AUTHOR. | REFERENCE. | CASES. | STAGE. | PERCENTAGE CURED. |
|---|---|---|---|---|
| Collected by—<br>Pottenger ... ... | *Therap. Gaz.*, 1903, xxvii. 163. | 611 | I. | 64 |

Denys[2] treated 174 cases by tuberculin alone without special hygienic treatment; 29 per cent. were cured and 42 per cent. greatly improved. Turban[3] has constructed a table

[1] Urwick, *Brit. M. J.*, 1905, ii. 172.

[2] Denys, quoted by Bosanquet, *op. cit.* [3] Turban, *Ibid.*

to show that resistance is permanently heightened by tuberculin. He treated 152 cases in the second stage without tuberculin, of which 70 per cent. were alive four years after; while of 48 treated with tuberculin 75 per cent. were alive. In the third stage, of 84 cases treated without tuberculin 50 per cent. were alive in two years, and of 21 treated with tuberculin 75 per cent. Grünbaum[1] has obtained satisfactory results with Koch's bacterial emulsion, and A. E. Wright[2] has reported similarly with TR. In cases of lupus Malcolm Morris[3] has recently stated that he considers it a useful adjunct to other treatment, and Neisser,[4] who has used the old tuberculin since its introduction, still believes in its value. Sir T. Anderson[5] in Dublin has used it with success when other measures have failed. Altogether I have found records of 65 cases, 58 of which were greatly improved or cured, 4 were partly cured, 2 slightly improved, and in 1 there was no improvement. Gerhardt and Senator[6] are of opinion that the old tuberculin is useful in tuberculous laryngitis, and recently Wright[7] and Hurry Fenwick have reported successful results with Koch's new tuberculin (emulsion) in cases of urinary tuberculosis. Warren Brown[8] has found TR valuable in localised tuberculous cystitis. Anderson[9] has published two cases of tuberculous peritonitis in children which were treated with old tuberculin, and were discharged well in three months; and also one case of tuberculous adenitis and one of Addison's disease in which very good results were obtained. Most of the published results refer to Koch's TR or his emulsion ; but some success has apparently attended the less known preparations, such as Béraneck's.[10] In March, 1904, 60 out of 90 cases were said to have been improved; but in general these products have not

[1] Grünbaum, *Lancet*, 1904, ii. 886.

[2] Wright, West Kent Med.-Chir. Soc., April 8th, 1904 ; *Lancet*, 1904, i. 1127 ; *Clin. J.*, 1904-05, xxv. 54.

[3] Malcolm Morris, *Lancet*, 1904, ii. 1132.

[4] Low, *Scottish M. and S. J.*, 1905, xvi. 397.

[5] Anderson, *Brit. J. Dermat.*, 1905, xvii. 317.

[6] Gerhardt and Senator, quoted by Bosanquet, *op. cit.*

[7] Wright and Hurry Fenwick, *Lancet*, 1904, i. 935.

[8] Warren Brown, *Brit. M. J.*, 1905, i. 1089. [9] Anderson, *loc. cit.*

[10] Béraneck, *loc. cit.*

been used sufficiently long for satisfactory statistical deductions to be made as to their value.

III. The limitations to the value of tuberculin are important, as no doubt ignorance of these, as well as improper usage, was the cause of the failure of Koch's first preparation. They may briefly be stated as follows:—

(1) Tuberculin is useless where the protective mechanism is worn out, as in advanced cases, and cannot respond to the stimulus of an active immuniser. This will be true whatever view is taken of the physiological action of the remedy.

(2) It is of little use in mixed infections, where pyogenic cocci are responsible for most of the symptoms.

(3) Koch's old tuberculin and similar preparations are contra-indicated in deep-seated lesions where the necrotic material formed by the local reaction cannot escape. It is uncertain, however, whether there is any actual danger of spreading the disease by liberating tubercle bacilli in the tissues.

(4) It has been observed that any considerable degree of fever contra-indicates Koch's old tuberculin, but not the bacterial emulsion.

*Anti-Tuberculous Sera.*—The earliest attempt to produce a passive immunity was that of Harricourt and Richet in 1890.[1] It was not successful, as they used the serum of a naturally immune animal, which is now known to have no anti-toxic power. Hirschfelder, in 1897,[2] introduced a substance named oxytuberculin. This, though prepared from tuberculin, is intended to act as an anti-toxin, and should thus be classed with the sera as a passive immuniser. It consists of tuberculin oxidised with $H_2O_2$, and is sterilised before use. Macfarland[3] in the same year tried the serum of an ass which had been immunised with tuberculin on fifteen cases of tuberculosis without definite result. Nicholls (1904), using Koch's TR as an immuniser, obtained a serum, but it was practically of little value. Fisch,[4] in the United

[1] Harricourt and Richet, *Compt. rend. Soc. de Biol.*, 1890, p. 310.

[2] Hirschfelder, quoted in *Brit. M. J.*, 1905, ii. 966.

[3] Macfarland, *J. Am. M. Ass.*, 1897, xxix. 359.

[4] Fisch, *J. Am. M. Ass.*, 1897, xxix. 882; 1898, xxx. 303; 1899, xxxii. 73.

States, produced a serum in 1898 prepared from horses injected with Koch's TR. I have only found records of 31 cases, of which 24 were considerably improved, but it is said to have been largely used in the United States.[1]

Maragliano,[2] in 1899, prepared a serum by injecting horses with a mixture of an aqueous extract of the bacilli and a preparation of toxalbumens from liquid cultures. The serum of the horses neutralises tuberculin, and is said to produce bacteriolysis *in vitro*. 2,900 cases have been published: in circumscribed apyretic cases 38 per cent. were cured, in circumscribed febrile cases 18 per cent., in diffuse tuberculous bronchitis 14 per cent., and in cases with cavities 5.6 per cent. These results, however, have not been confirmed by observers in other countries.

Marmorek,[3] in 1903, described a new serum, which has since been tried on a number of cases in this country and elsewhere. I have already alluded to his view that Koch's tuberculin is not the true toxin of tuberculosis, but merely a sensitiser of the bacilli. Acting on this idea, he made cultivations of a special strain of tubercle bacilli ("primitive" bacilli), which produce much less tuberculin than ordinary cultures, and differ morphologically from the full-grown type. Grown on special culture media, which are really unfavourable soil for the ordinary growth of the bacilli, this strain of "primitives" can be induced to form a new toxic substance, which Marmorek regards as the real toxin of the disease. He states that he has immunised experimental animals with this toxin, and that their serum contains anti-toxins. He admits that his results have not been quite constant, and that the toxin might with advantage be more powerful. Marmorek wished to test his serum on the most hopeless cases first, and then, if it failed here, to gradually try it in less and less severe cases, in order to find at what point its efficacy could first be detected clinically. His early results were much criticised in Paris. Dieulafoy[4] stated that he had

[1] Larned, *Therap. Gaz.*, 1905, xxix. 582.

[2] Maragliano, *Berlin. klin. Wchnschr.*, 1899, p. 1,073; *also* Cattle, *Practitioner*, 1905, lxxiv. 518.

[3] Marmorek, *loc. cit.*, and *Lancet*, 1905, ii. 1280.

[4] Dieulafoy, *Semaine Méd.*, Dec. 2nd, 1903.

tried the serum in 7 cases with no good result, and that he had failed to immunise guinea pigs with it. Hallopeau[1] was equally positive that it did no good to 7 patients, and in 2 he observed obstinate ulcerating nodules at the site of injection, which he believed were due to the toxins in the serum. Monod,[2] on the other hand, reported good results in glandular and localised cases. Latham, who has tried it in pulmonary cases, believes that it does good in some cases, and at the recent congress in Paris twelve observers spoke in its favour. It is apparently useless in very advanced cases of phthisis, in miliary tuberculosis and meningitis, and in mixed infections, but should be tried in moderately advanced phthisis and in tuberculous diseases of bones and joints, fistulæ and ulcers, and tuberculous inflammation of serous membranes. Marmorek states that it has done good in about three-fourths of the cases in which it has been tried, and that some of the failures occurred in patients who were *in extremis*. Others, again, are intolerant of serum-therapy. Much appears to depend on the mode of administration and the general technique; it is admitted that the anti-toxic power is not high, and that no standardisation of the serum is as yet possible.

Stephani,[3] who quotes no figures, says that out of a large number of cases distinct improvement occurred in half, symptomatic improvement in three-fifths, and in five cases rapidly advancing disease was checked. Habershon[4] stated that in certain moderately bad cases it had produced distinct improvement, but no actual cures had been observed at the Brompton Hospital. Levin[5] reported on 156 cases treated in Sweden with on the whole encouraging results (75 per cent. improved). He also confirmed to a certain extent Marmorek's animal experiments, but thought the serum too weak to produce absolute immunity. Most of the published reports refer to only small groups of three or four cases, and as thus of little value statistically, though they seem to indicate the usefulness of the treatment.[6]

1 Hallopeau, *Ibid.* 2 Monod, *Ibid.* 3 Stephani, *Lancet*, 1905, ii. 1281.

4 Habershon, *Ibid.* 5 Levin, *Ibid.*, p. 1282.

6 *Vide* Bassano, *Ibid.*, p. 760; *also* Latham, *Pulmonary Consumption*, 2nd Edition, 1905.

Speaking generally, it would seem that serum-therapy should be useful in cases when there is little response to active immunisation to toxins, that its danger lies mainly in over-dosage, whereby an excess of anti-toxin (amboceptors or copula) is acquired, and that the principal causes of failure are: (1) The small amount of circulating toxin in tuberculous disease; (2) the rapid elimination of anti-toxins; (3) the low anti-toxic valency of the serum of injected animals.

Perhaps, in conclusion, I may say a few words on the subject of Behring's recent announcement, because his method, though only known to us at present in outline, may in the near future become an important factor in all discussions on immunisation against tuberculosis. As far as I am able to understand it the method is this: Behring first separates certain toxic and useless principles from the bacilli, leaving residual forms which he subsequently reduces to an amorphous powder. This powder is, or contains, a substance called TC. It is not an anti-toxin, but the precursor of a substance TX formed by it in combination with lymphoid cells in the body. The formation of TX coincides with the production of eosinophil cells and the condition of immunity. Behring's process is thus not an active immunisation, because he does not inject a toxin in order to stimulate the production of anti-toxins. It is in a sense a passive immunisation, because he injects the substance which produces the reaction to Koch's tuberculin, and which the organism in any natural attempt to acquire immunity has to make for itself. But it differs from former methods of passive immunisation in that no directly anti-toxic animal serum is used. The main difficulty which an infected animal has is to get this TC from the bacilli. If he can get enough TC, this combines with the lymphoid cells to produce TX, and the animal becomes immune. Behring's process supplies unlimited quantities of TC ready made, and it is apparently to be used more or less as a prophylactic. TC can produce tubercles which neither caseate nor soften, which must thus be regarded as part of the natural protective reaction against the bacterial invasion.

